# Radiomics in Action: Multimodal Synergies for Imaging Biomarkers

**DOI:** 10.3390/bioengineering12111139

**Published:** 2025-10-22

**Authors:** Everton Flaiban, Kaan Orhan, Bianca Costa Gonçalves, Sérgio Lúcio Pereira de Castro Lopes, Andre Luiz Ferreira Costa

**Affiliations:** 1 Postgraduate Program in Dentistry, Dentomaxillofacial Radiology and Imaging Laboratory, Cruzeiro do Sul University (UNICSUL), São Paulo 01506-000, Brazil; evertonflaiban@gmail.com; 2 Department of Dentomaxillofacial Radiology, Faculty of Dentistry, Ankara University, Ankara 06560, Turkey; knorhan@dentistry.ankara.edu.tr; 3 Department of Diagnosis and Surgery, The Institute of Sciences and Technology of São Paulo State University (UNESP), São José dos Campos 12245-000, Brazil; bianca.goncalves@unesp.br (B.C.G.); sergio.lopes@unesp.br (S.L.P.d.C.L.); 4 Department of Anesthesiology, Oncology and Radiology, Faculty of Medical Sciences, University of Campinas (UNICAMP), Campinas 13083-887, Brazil

**Keywords:** artificial intelligence, cone beam computed tomography, imaging biomarkers, quantitative imaging, radiomics

## Abstract

Radiomics has recently begun as a transformative approach in medical imaging, shifting radiology from qualitative description to quantitative analysis. By extracting high-throughput features from CT (Computed Tomography), MRI (Magnetic Resonance Imaging), PET/CT (Positron Emission Tomography/Computed Tomography), and CBCT (Cone Beam Computed Tomography), radiomics enables the characterization of tissue heterogeneity and the development of imaging biomarkers with diagnostic, prognostic, and predictive values. This narrative review explores the historical evolution of radiomics and its methodological foundations, including acquisition, segmentation, feature extraction and modeling, and platforms supporting these workflows. Clinical applications are highlighted in oncology, cardiology, neurology, and musculoskeletal and dentomaxillofacial imaging. Despite being promising, radiomics faces challenges related to standardization, reproducibility, PACS/RIS (Picture Archiving and Communication System/Radiology Information System) integration and interpretability. Professional initiatives, such as the Image Biomarker Standardization Initiative (IBSI) and guidelines from radiological societies, are addressing these barriers by promoting harmonization and clinical translation. The ultimate vision is a radiomics-augmented radiology report in which validated biomarkers and predictive signatures complement conventional findings, thus enhancing objectivity, reproducibility, and advancing precision medicine.

## 1. Introduction

Over the past two decades, the progressive digitalization of medical imaging has catalyzed a change from qualitative visual interpretation to quantitative image analysis [1]. This evolution coincides with the rapid development of artificial intelligence (AI) methods, which are increasingly integrated into clinical research and practice to support diagnostic, prognostic and therapeutic decisions [2]. One of the most compelling developments at the intersection of imaging and data science is radiomics, a field which seeks to convert medical images into mineable data through the extraction of high-throughput quantitative features [1,3,4,5,6].

The term “radiomics” was formally introduced in the early 2010s, particularly with the pioneering work of Lambin et al. [3] in oncology, in which imaging features were shown to correlate with tumor phenotype and patient outcomes. However, the conceptual foundations of radiomics trace back to earlier efforts in texture analysis and pattern recognition in radiology, dating to the late 20th century [7]. What distinguishes radiomics is its systematic, high-dimensional and data-driven approach, that is, it assumes that medical images contain latent biological information imperceptible to the human eye, but which can be revealed through mathematical modeling [1,3,4,5,8].

By quantifying image heterogeneity (through first-order statistics, textural matrices, and transform-based descriptors), radiomics aims to provide objective, reproducible and clinically relevant biomarkers [3,9,10]. These features can be extracted from multiple imaging modalities, most notably computed tomography (CT) and magnetic resonance imaging (MRI), which offer complementary anatomical and functional information. More recently, cone beam computed tomography (CBCT) has also emerged as a promising modality in radiomics for maxillofacial diagnosis [11,12]. In parallel, positron emission tomography combined with computed tomography (PET/CT) has become a central platform in oncologic radiomics, in which the integration of metabolic and anatomical information allows characterization of tumor heterogeneity, prediction of treatment response, and assessment of survival outcomes [8,9,13].

Although initially focused on oncology, radiomics has expanded to diverse clinical domains, including neurology, cardiology, musculoskeletal imaging, and head and neck surgery [13,14,15,16].

Despite being promising, radiomics still faces inherent challenges. Issues such as standardization, reproducibility, and clinical integration have limited its application in daily practice. Moreover, the majority of radiomic models are retrospectively constructed, not being externally validated and relying on handcrafted features whose biological interpretation remains unknown.

This narrative review aims to explore how radiomics has evolved from a conceptual framework to a practical tool in medical imaging, highlighting its historical development, methodological principles, and clinical applications, particularly in CT, PET/CT, CBCT and MRI. By integrating foundational studies, technical guidelines and recent advances, one seeks to provide a critical and cohesive narrative reflecting the current state of the field and its potential to transform diagnostic imaging.

## 2. Narrative Review Process

This article is a narrative review aimed at providing a broad and integrative perspective on radiomics in medical imaging, with particular emphasis on its historical emergence, conceptual underpinnings, methodological framework, and clinical applications in CT, CBCT, PET/CT and MRI. In line with the exploratory nature of narrative reviews, no formal systematic protocols (e.g., PRISMA) were applied.

Relevant literature was identified through targeted and iterative searches of electronic databases, including Web of Science, PubMed, Scopus, and Google Scholar. The keyword combinations used in the search strategy included the following: “radiomics”, “texture analysis”, “quantitative imaging,” “computed tomography”, “cone beam computed tomography”, “magnetic resonance imaging”, and “artificial intelligence”. No restrictions regarding publication date or study type were imposed.

Studies were selected based on their conceptual relevance, methodological rigor, historical significance, or clinical impact. Emphasis was placed on seminal publications, high-impact reviews, technical guidelines (e.g., Image Biomarker Standardization Initiative—IBSI), and recent original studies exemplifying key trends or challenges in the field. Although the review does not claim to be exhaustive, it seeks to synthesize the most influential contributions shaping the development and current landscape of radiomics in cross-sectional imaging.

## 3. The Origins and Conceptual Foundations of Radiomics

The roots of radiomics are deeply embedded in the evolution of texture analysis and pattern recognition techniques developed in the late 20th century, particularly for applications in computer-aided diagnosis (CAD) [17]. Early studies explored how quantitative measures derived from medical images, such as gray-level distributions and spatial relationships between pixels, could reveal underlying tissue characteristics invisible to the naked eye [18,19,20]. These pioneering efforts laid the groundwork for what would later become known as feature-based image analysis.

**Figure 1:**
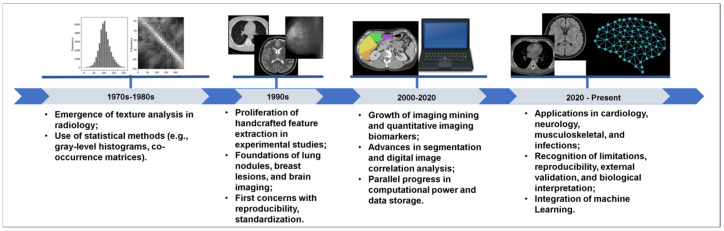
Key milestones in the history of radiomics highlighting the evolution from early texture analysis and pattern recognition in the 1970s to the integration of deep learning and regulatory efforts in the 2020s.

During the 1980s and 1990s, several researchers began to experiment with statistical texture descriptors, including co-occurrence matrices, run-length features, and fractal-based metrics, to differentiate benign from malignant lesions, particularly the imaging of breast, lung nodules, and brain tumors [21,22,23]. However, these approaches were often limited by computational constraints, heterogeneous imaging protocols and absence of standardized feature definitions. Despite their technical promise, they remained largely within academic environments, with limited clinical translation. Figure 1 illustrates key milestones in the historical development of radiomics, moving from early texture analysis to modern AI-integrated workflows.

The conceptual leap toward radiomics occurred when these fragmented methods were unified into a coherent framework treating medical images not merely as pictures for visual inspection, but as quantitative datasets. The introduction of the term “radiomics”, notably by Lambin & colleagues in 2012 [5], marked a turning point in this trajectory. Their work demonstrated that high-throughput image features could be systematically extracted from routine clinical imaging, being linked to tumor genotype and phenotype and patient outcomes in oncology. This model shift established radiomics as a connection between medical imaging and personalized medicine.

What distinguishes radiomics from its precursors is its systematic, high-dimensional and data-driven approach [1,3,5]. Unlike traditional visual interpretation or qualitative scoring systems, radiomics enables the extraction of hundreds to thousands of features capturing subtle variations in intensity, texture, shape, and wavelet transformations [1,5,24]. These features are often described as “handcrafted,” as they are based on predefined mathematical formulas [1,3,5,24,25]. They aim to quantify image heterogeneity, which is increasingly recognized as a surrogate marker for biological complexity, especially in cancer and inflammatory diseases [26].

Particularly, radiomics is not merely a tool for image feature extraction, as it is a comprehensive analytical pipeline integrating image preprocessing, segmentation, feature computation, selection, and predictive modeling [5]. The basic goal is to provide clinically meaningful and reproducible biomarkers which can aid in diagnosis, prognostication and treatment planning [3,5].

In summary, the conceptual foundation of radiomics is rooted in decades of exploratory work in image analysis and has since matured into a formalized discipline. Its development reflects a broader trend in medicine, namely: the transformation of qualitative disciplines into a data-rich, quantitative approach [5,27]. This evolution continues to accelerate as advances in computational power, machine learning, and imaging standardization further unlock the potential of medical images as sources of phenotypic information.

## 4. Image Acquisition and Standardization

The radiomics workflow begins with image acquisition, a critical step in which clinical feasibility should be balanced with data fidelity to directly influence the quality, reproducibility, and clinical applicability of the extracted features [9,28]. Radiomics is highly sensitive to variations in scanner type, acquisition protocol, voxel resolution, and reconstruction algorithms, as well as other parameters such as filters, contrast agent use, field strength and pulse sequence for MRI, and scanner models. These factors can introduce subtle yet meaningful changes in feature values, thus potentially impairing the reproducibility across studies and confounding the modeling efforts [27,29,30].

Although early radiomic studies focused predominantly on computed tomography (CT), such as the landmark work by Aerts et al. [31], the methodology has since been extended to magnetic resonance imaging (MRI), positron emission tomography (PET), and, more recently, cone beam computed tomography (CBCT). Each modality offers unique opportunities and challenges in radiomic analysis. CT provides calibrated intensity values (in Hounsfield units), enabling robust comparisons across scanners. MRI, on the other hand, offers superior soft tissue contrast, but lacks standard intensity scale, leading to greater variability. PET adds functional and metabolic information to structural imaging, although its lower spatial resolution and sensitivity to motion require careful handling [32].

CBCT has recently gained traction in dental and maxillofacial radiomics due to its accessibility and ability to visualize bone structures with high spatial resolution and low radiation dose. It has been successfully used to diagnose jaw cysts by using machine learning [33], estimate biological age from mandibular condyles [11], and evaluate condylar resorption after orthognathic surgery [34]. However, CBCT is particularly susceptible to noise, scatter artifacts and non-uniform voxel intensities, which may impair feature stability.

Standardization strategies are essential across all modalities to mitigate these issues. These include protocol harmonization, which involves the use of uniform acquisition settings to reduce inter-scan variability, particularly important in multicenter studies [29]. Resampling techniques are commonly used to adjust voxel dimensions and image matrix sizes, thus ensuring consistency in feature extraction. Intensity normalization methods, such as histogram matching, z-score normalization, and Nyúl scaling, are particularly useful in addressing signal variability in modalities like MRI and CBCT [9,35]. Additionally, phantom-based calibration can be employed to assess scanner performance and minimize technical variation in radiomic feature distributions [36].

In retrospective studies and multicenter datasets, statistical techniques (e.g., ComBat harmonization) were shown to be promising to adjust batch effects while preserving biologically relevant signals [37].

It is important to note that the choice of modality should be driven by clinical context. For example, MRI-based radiomics has shown to be promising in the characterization of head and neck tumors, enabling the extraction of predictive features related to tumor hypoxia and HPV status [38]. Similarly, CT and MRI radiomics have been applied to the evaluation of bone and soft-tissue sarcomas, although reproducibility and validation strategies remain areas of active investigation [39].

As radiomics advances toward clinical adoption, the need for modality-specific preprocessing pipelines and community-driven standards becomes increasingly urgent. Initiatives, such as the IBSI, play a decisive role in defining consensus guidelines for feature extraction and image processing, enabling radiomics to progress from research to routine care [40,41,42].

As for CBCT, standardization poses challenges due to greater susceptibility to scatter, beam hardening, and machine-specific reconstruction variations. Recent studies in dentomaxillofacial radiology emphasize the need for harmonized acquisition settings and preprocessing pipelines for CBCT-based radiomics to be clinically viable [33,43].

## 5. Segmentation: ROI and VOI Strategies

Segmentation defines the spatial region from which radiomic features are extracted and can be performed manually, semi-automatically, or automatically. The two principal approaches include strategies involving region-of-interest (ROI) and volume-of-interest (VOI). ROI typically involves delineating a 2D slice, often chosen for its representative anatomy or pathology. Although this method is time-efficient, it may underrepresent the lesion heterogeneity [8,9,44].

VOI segmentation, in contrast, captures the entire 3D volume of the lesion or organ, enabling more comprehensive quantification of spatial texture and shape descriptors [8,9,44]. Although more computationally intensive, VOI is favored in radiomic studies for its robustness and ability to reflect intralesional heterogeneity [44].

However, a major drawback of radiomics is the labor-intensive process of manual image segmentation, which typically requires a trained imaging specialist to delineate structures on each image slice individually [24,27,44].

Semi-automated segmentation has been reported to enhance the consistency and reliability of specific radiomic features. Nevertheless, both manual and semi-automated approaches remain vulnerable to notable inter-observer variability, particularly when dealing with lesions showing poorly defined margins, which can lead to unstable feature extraction [24,44].

Automated segmentation using AI models, especially U-Net architectures, has shown to be promising in increasing reproducibility, although manual correction remains necessary in many clinical applications [45,46,47].

## 6. Feature Extraction: First-Order, Texture, and Transform-Based Features

Following segmentation, the selected ROI or VOI undergo quantitative analysis through the extraction of a diverse set of radiomic features. These descriptors are typically organized into three main categories, each capturing complementary aspects of the underlying image data [9].

First-order features summarize the distribution of voxel (or pixel) intensities within the defined area, without considering spatial relationships. Common parameters include measures of central tendency (mean, median), dispersion (variance, standard deviation), histogram shape (skewness, kurtosis), and overall signal heterogeneity (entropy) [9].

Texture features extend beyond the basic intensity statistics to characterize spatial patterns and arrangement of gray levels within the structure. Derived from statistical matrices, such as the gray-level co-occurrence matrix (GLCM), gray-level run length matrix (GLRLM), gray-level size zone matrix (GLSZM), and neighborhood gray-tone difference matrix (NGTDM), they capture properties like homogeneity, contrast, correlation, and complexity [9,48]. Texture features are especially valuable for quantifying intralesional heterogeneity, which has been associated with tumor aggressiveness, fibrosis patterns, and inflammatory processes [1,9].

Transform-based features are obtained after applying mathematical filters or transforms to the image data, such as wavelet decomposition, Laplacian of Gaussian (LoG), or Gabor filters [6,9]. These methods emphasize different spatial frequency components or edge characteristics, enabling the detection of subtle textural variations at multiple scales. By integrating these multi-scale descriptors, radiomics can uncover microstructural information which may correlate with histopathological or molecular profiles [1].

As for CBCT, recent research in dentomaxillofacial radiology has shown that texture and transform-based features, when combined with machine learning classifiers, can differentiate cystic lesions of the jaw with high accuracy [33] and estimate age based on morphological and structural patterns of the mandibular condyle [11]. Beyond these applications, radiomics is also showing promise in clinically relevant scenarios such as distinguishing cystic lesions from tumors, assessing temporomandibular joint (TMJ) disorders, and monitoring bone healing in longitudinal follow-up of periapical and surgical cases. However, due to the CBCT higher sensibility to scattering, beam hardening, and non-standardized intensity scales, rigorous preprocessing, and normalization are essential to ensure feature robustness [49,50,51,52,53].

As for CT, these feature categories have been extensively used to characterize oncologic lesions, assess treatment response, and stratify patient prognosis [8,40]. CT calibrated Hounsfield units provide a reliable foundation for quantitative analysis, although variations in acquisition and reconstruction protocols still require harmonization to maintain the reproducibility of the features [9].

As for MRI, radiomic features have been successfully extracted from both anatomical (e.g., T1-weighted, T2-weighted) and functional sequences (e.g., diffusion-weighted imaging, perfusion MRI) [54,55]. Although MRI offers superior soft-tissue contrast and multiparametric capabilities, the intensity non-standardization between scanners and protocols poses a challenge which must be addressed with robust normalization strategies [1,9].

By combining first-order, texture, and transform-based features from these modalities, each with distinct strengths, radiomics enables a comprehensive, multi-parametric representation of the tissue architecture and heterogeneity, thus paving the way for the development of reproducible and clinically relevant imaging biomarkers [1,8,9].

**Figure 2:**
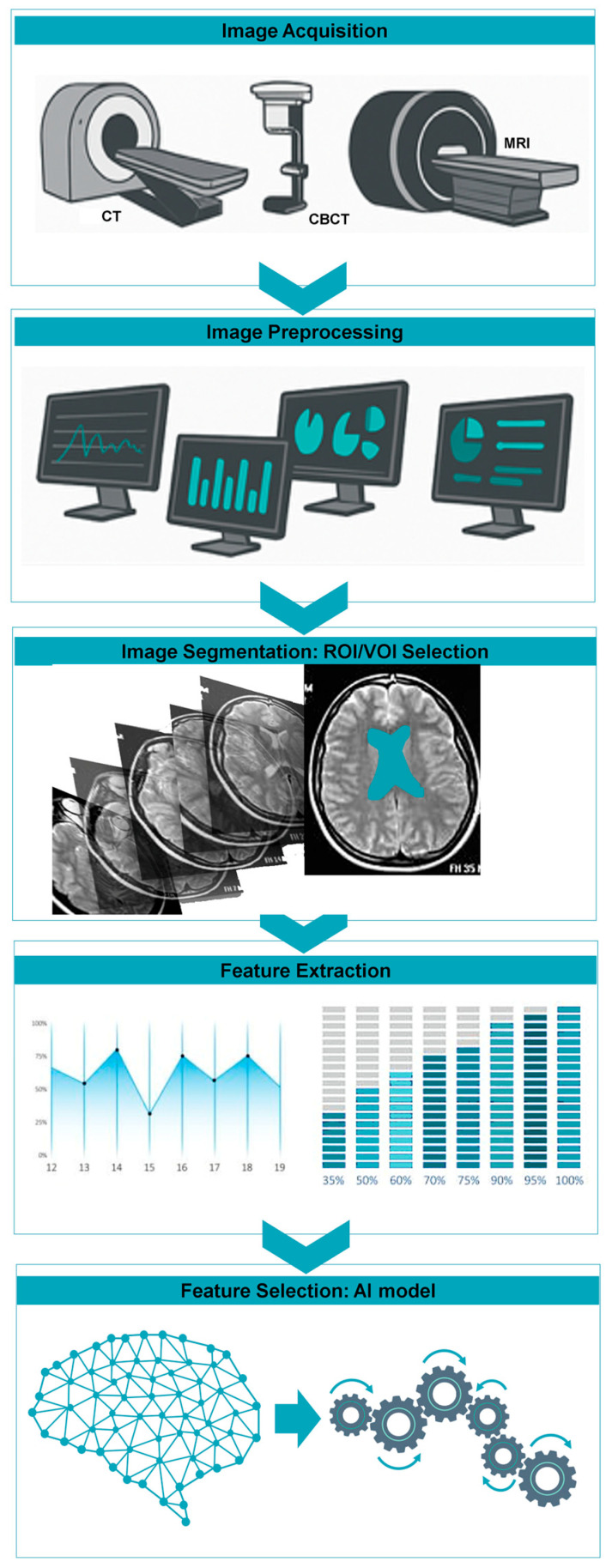
Radiomics workflow: from medical image acquisition and preprocessing to segmentation, feature extraction, selection, and model building through AI for clinical decision support.

An overview of the radiomic workflow (segmentation-to-feature-extraction) is illustrated in Figure 2, highlighting the integration of AI-based approaches in the final modeling stage.

## 7. Feature Selection, Dimensionality Reduction, and Model Building

### 7.1. Feature Selection and Dimensionality Reduction

**Figure 3:**
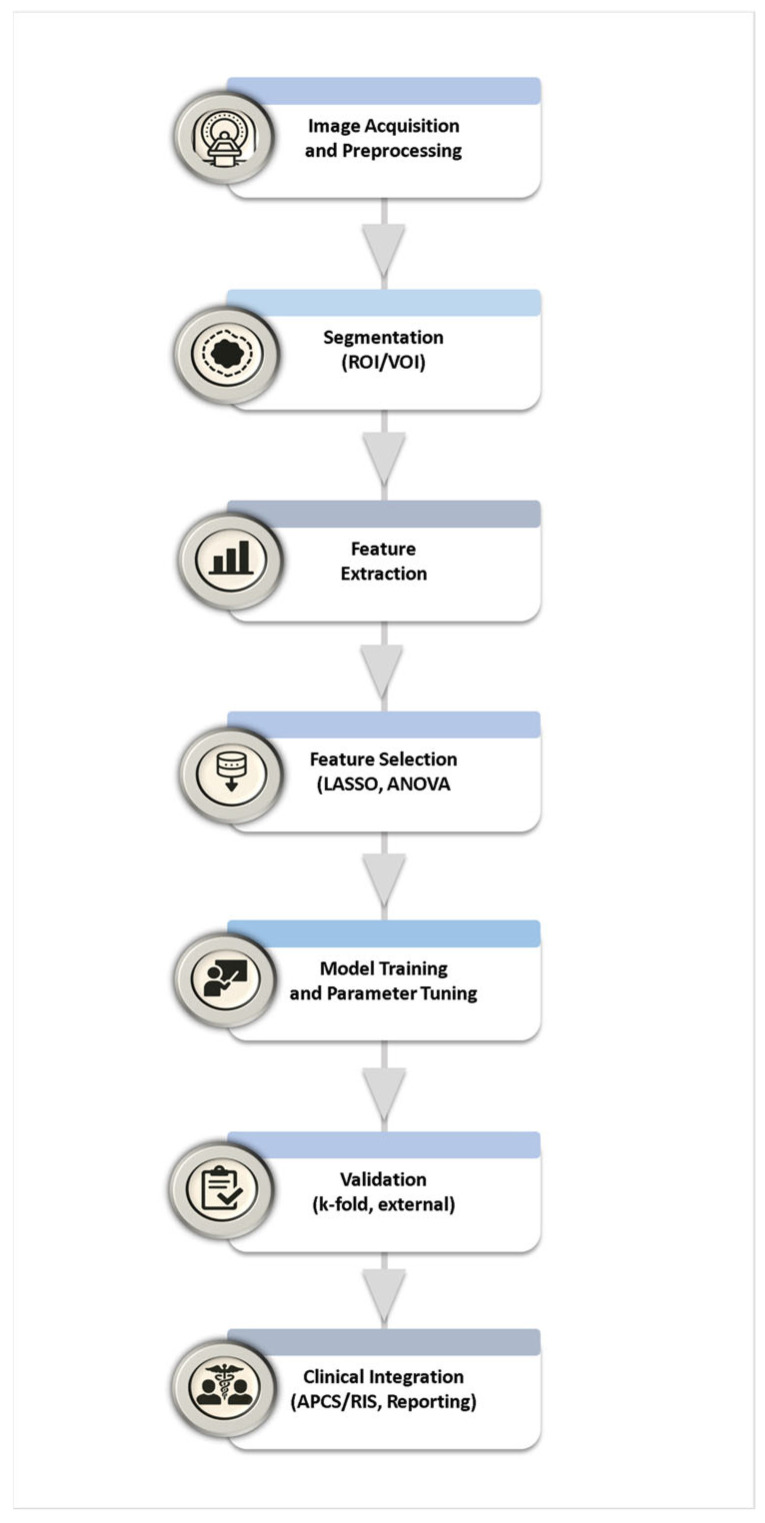
Radiomics experimental workflow: Overview of the complete radiomics pipeline, from image acquisition and preprocessing to clinical integration. The diagram summarizes key stages including segmentation of regions or volumes of interest (ROI/VOI), feature extraction, selection, model training, validation, and incorporation of radiomics-based biomarkers into PACS/RIS and structured radiology reporting.

Radiomic datasets are typically high-dimensional, containing hundreds or even thousands of features derived from a single imaging study [1,56]. Although this has the potential to capture subtle biological heterogeneity, it however also introduces redundancy, noise, and a substantial risk of overfitting, particularly when the number of patients is limited compared to the number of features [56]. To address these challenges, feature selection and dimensionality reduction are indispensable steps in the radiomics workflow. The overall workflow of feature selection, dimensionality reduction, model building, and validation is summarized in Figure 3.

### 7.2. Feature Selection

Feature selection aims to identify the subset of features which are most informative for a given clinical outcome, and at the same time, to eliminate redundant or irrelevant variables [56,57,58,59]. This process improves model stability, enhances interpretability, and reduces computational complexity. Common strategies are broadly categorized into three groups [42,59] as follows:

(a) Filter methods: These rely on statistical measures which are independent of the predictive model, such as analysis of variance (ANOVA), Pearson’s correlation, or mutual information. They are computationally efficient and suitable for initial screening, although they may overlook complex interdependencies among features.
(b) Wrapper methods: These approaches iteratively evaluate subsets of features in conjunction with machine learning algorithms. The recursive feature elimination (RFE) method is one widely used example, which progressively discards the least informative variables until the optimal subset is achieved. Although wrappers are powerful in capturing feature interactions, they are computationally expensive and prone to overfitting in limited datasets.
(c) Embedded methods: These integrate feature selection into the model training process itself. Penalized regression methods, such as LASSO (least absolute shrinkage and selection operator) and elastic net, are among the most frequently used in radiomics. These techniques simultaneously select features and assign weights, yielding parsimonious models which balance predictive performance with interpretability.

**Figure 4:**
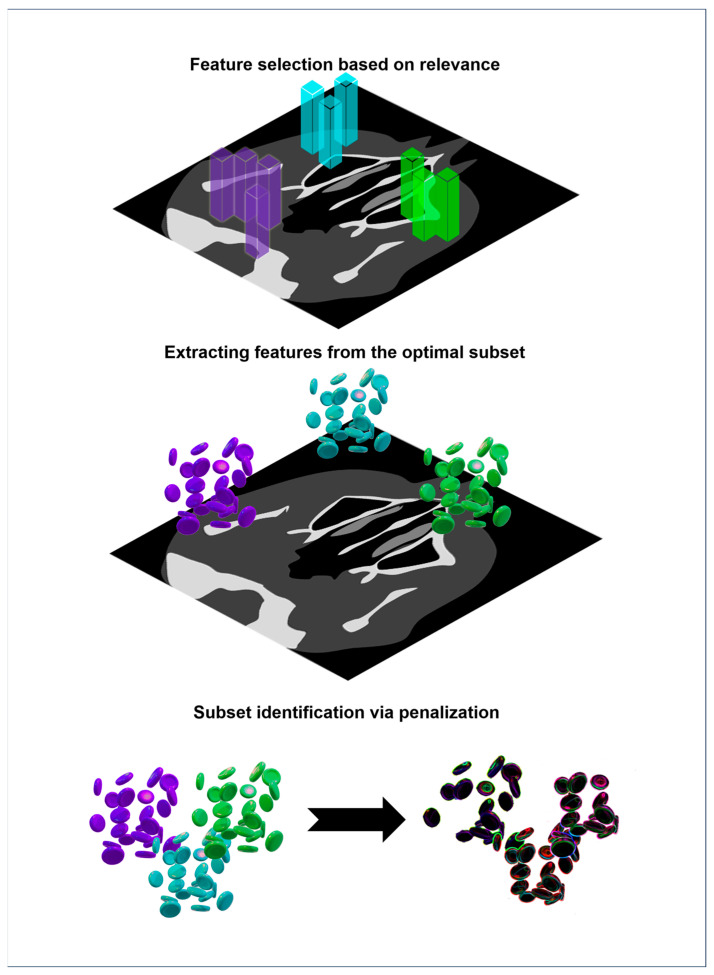
Workflow of feature selection and optimization in radiomics illustrating the sequential steps from initial feature extraction to refinement of predictive models. The process encompasses different strategies of feature selection (filter, wrapper and embedded methods), followed by model optimization and validation to ensure reproducibility and robustness of radiomic biomarkers.

In radiomics, embedded approaches are often favored due to the high dimensionality and relatively small sample size of most datasets. LASSO regression has become the most commonly used technique for stabilizing predictive modeling in oncology and beyond [59]. The overall workflow of feature selection, dimensionality reduction, model building, and validation is summarized in Figure 4.

### 7.3. Model Validation in Radiomics

In radiomics research, reproducibility depends heavily on rigorous methodological design. Data preprocessing typically includes voxel resampling, intensity normalization, and discretization of gray levels to ensure comparability across scanners and protocols. During model training, feature subsets selected through statistical or embedded methods (e.g., LASSO, random forests) are used to train predictive algorithms. Parameter tuning is performed using grid search or cross-validation to optimize hyperparameters and avoid overfitting. For validation, both internal (e.g., k-fold or bootstrapping) and external (independent dataset) approaches are recommended to assess generalizability. Addressing potential class imbalance, through resampling or weighted loss functions, is fundamental to maintain fairness and prevent biased model performance. Together, these steps define a transparent and reproducible framework for developing radiomics-based predictive models [56,57,58,59,60,61,62,63,64,65,66,67].

Once feature selection is complete, the resulting models must be subjected to rigorous validation to establish their reliability, reproducibility, and clinical utility [57,60]. Validation is not a single step, but a structured process ensuring that the model is not limited to the dataset on which it was trained and its predictive performance evaluated. Internal validation is typically the first stage, in which methods such as k-fold cross-validation, leave-one-out cross-validation, or bootstrapping are employed to estimate model stability and guard against overfitting [59,60,61]. These approaches repeatedly segment the available data into training and testing subsets, thus assessing how consistent the model’s predictions are across different samples of the same cohort [61,68,69].

Although internal validation provides valuable insight into robustness, it cannot fully guarantee that a model will generalize to unseen populations. For this reason, external validation is considered the gold standard. In external validation, the trained model is tested on an entirely independent dataset, ideally collected at different institutions, with distinct imaging protocols, scanners, or patient demographics. Such heterogeneity more accurately reflects real-world clinical variability and thus provides the most stringent assessment of generalizability. Models maintaining strong predictive performance under these conditions are far more likely to be translatable into clinical practice.

Beyond simple performance metrics, such as accuracy or AUC (Area under the Curve), validation in radiomics also involves assessing calibration, clinical usefulness, and reproducibility of feature extraction [59,60,61]. Multi-center studies, prospective trial designs and harmonization strategies for imaging protocols further strengthen the credibility of validated radiomic models [59]. Finally, only through this layered and systematic evaluation can radiomics evolve from exploratory image-based data mining (IBDM) into a rigorous framework for quantitative imaging biomarkers [8]. When robustly validated, radiomic signatures can support precision medicine by enabling non-invasive phenotyping, predicting treatment response and facilitating patient stratification in ways which complement or even surpass traditional clinical and pathological markers [8,9].

Recent developments in deep learning have transformed radiomics by enabling the automatic extraction of hierarchical image representations that capture complex spatial and contextual patterns beyond handcrafted features. Convolutional neural networks (CNNs) and attention-based models have demonstrated superior performance in tumor classification, treatment response prediction, and survival modeling compared with classical feature-based approaches [62,63]. In addition, hybrid AI–radiomics frameworks, which integrate deep features with handcrafted descriptors or clinical variables, are emerging as robust strategies that combine interpretability and predictive power [62,67,70]. Despite these advantages, deep learning models require large, balanced, and well-annotated datasets—posing a challenge known as data hunger—and often lack explainability, limiting clinical trust and regulatory approval. Current research is thus focusing on explainable AI (XAI) and transfer learning to mitigate these limitations and promote generalizable, interpretable models for real-world deployment [62,71,72].

Recent advances have introduced novel strategies to improve data diversity, robustness, and feature selection in radiomics and biomedical imaging. Generative Adversarial Networks (GANs) have been increasingly used for synthetic data augmentation, improving the training stability and performance of deep models in small or imbalanced datasets [73,74]. Such approaches can generate realistic lesion textures and reduce overfitting, complementing classical radiomics workflows. In parallel, metaheuristic feature selection methods, such as hybrid Artificial Bee Colony optimization combined with Adaptive LASSO, have shown superior ability to identify relevant features in high-dimensional datasets while controlling redundancy [75]. Together, these techniques represent a significant evolution toward more generalizable and data-efficient radiomics pipelines.

### 7.4. Statistical Evaluation of Model Performance and Robustness

In radiomics research, statistical analysis plays a central role in assessing the significance and robustness of extracted features and predictive models. Univariate and multivariate hypothesis testing, along with confidence intervals and non-parametric tests (e.g., Wilcoxon or Mann–Whitney), are frequently used to determine whether differences in model performance or feature distributions are statistically meaningful. Furthermore, bootstrapping and cross-validation are employed to estimate variability, while receiver operating characteristic (ROC) analysis and Area Under the Curve (AUC) confidence intervals quantify discriminative ability. These statistical approaches help ensure that observed results are not due to random chance, thereby reinforcing model credibility and clinical translatability [56,57,58,59,60,61,62,63,64,65,66,67].

## 8. Tools and Platforms: MaZda, PyRadiomics, LIFEx, MATLAB, and Others

A variety of open-source and commercial platforms are available to implement different stages of the radiomic pipeline, each with specific strengths and target applications. The choice depends on the imaging modality, research question, and desired level of flexibility for integration with preprocessing, segmentation, and machine learning workflows [5,9,59].

### 8.1. MaZda

One of the earliest and most widely cited platforms for texture analysis, MaZda offers a GUI-based environment facilitating feature extraction from 2D and 3D images. It includes classic radiomic descriptors such as gray-level co-occurrence matrix (GLCM) and run-length matrix (RLM) features, making it valuable for historical comparisons and educational purposes [68]. MaZda is often used where reproducibility of early texture methods is important and supports batch processing for large datasets.

### 8.2. PyRadiomics

This is a Python-based package compliant with the IBSI, with PyRadiomics being among the most widely used open-source tools in radiomic research [59,69]. It integrates with 3D Slicer for interactive segmentation and offers a broad range of feature extraction options, including first-order statistics, texture features (i.e., GLCM, GLRLM, GLSZM, NGTDM), and shape descriptors. PyRadiomics supports preprocessing (i.e., resampling, intensity normalization, filtering) and produces detailed metadata for reproducibility [69].

### 8.3. LIFEx

Designed primarily for PET/CT and MRI radiomics, LIFEx emphasizes standardization and reproducibility by providing built-in segmentation tools, integration of calibration phantoms, and IBSI-compliant feature definitions [76]. It is user-friendly and requires minimal programming skills, with direct DICOM (Digital Imaging and Communications in Medicine) import from PACS (Picture Archiving and Communication System) systems, making it suitable for multicenter studies.

### 8.4. MATLAB

Although not exclusively a radiomic platform, MATLAB is a powerful environment widely used for medical image analysis and radiomics research (4). Its toolboxes for image processing, statistics, and machine learning allow fully customized pipelines, from preprocessing to predictive modeling. MATLAB supports custom feature design (GLCM, GLRLM, wavelet-based features) and integrates with deep learning frameworks for hybrid AI–radiomic models.

### 8.5. Other Platforms

Several other tools are noteworthy, namely:

IBEX (Imaging Biomarker Explorer): Open-source, MATLAB-based, with multimodal analysis and custom feature extraction [77,78,79].

RaCaT: Specialized in batch processing and harmonization in multicenter studies [80].

CERR (Computational Environment for Radiotherapy Research): Focused on radiotherapy imaging datasets, integrating dose-volume histogram and radiomic analysis [81].

RadiomiX: Commercial, CE-marked software with quality assurance and control modules, suited for regulatory-grade studies [82].

Many of these platforms support batch processing, DICOM integration, phantom calibration, and direct linkage to machine learning frameworks, thus enabling end-to-end automation of the radiomic pipeline.

Table 1 [68,69,76,77,79,80,81] summarizes the main technical characteristics of the most widely used radiomic tools and platforms, including license type, supported imaging modalities, and segmentation approaches. Table 2 [68,69,76,77,79,80,81] provides a complementary overview of their key features, advantages, and limitations.

## 9. Clinical Applications in Cross-Sectional Imaging

This part of the review discusses the most relevant clinical applications of radiomics across cross-sectional imaging techniques, focusing first on oncology, then exploring advances beyond oncology, and finally discussing predictive, prognostic, and diagnostic modeling. Together, these perspectives highlight the potential of radiomics in connecting routine imaging data to precision medicine.

Yet, radiomics is not confined to oncology. With the growing accessibility of quantitative imaging pipelines, cross-sectional modalities such as CT, MRI, PET, and CBCT are increasingly being used in non-oncologic diseases. Emerging evidence supports its value not only in cardiology through the analysis of coronary plaques and myocardial tissue, but also in neurology, musculoskeletal disorders, and dentomaxillofacial imaging. These developments highlight the breadth of radiomics in characterizing tissue heterogeneity across organ systems.

**Figure 5:**
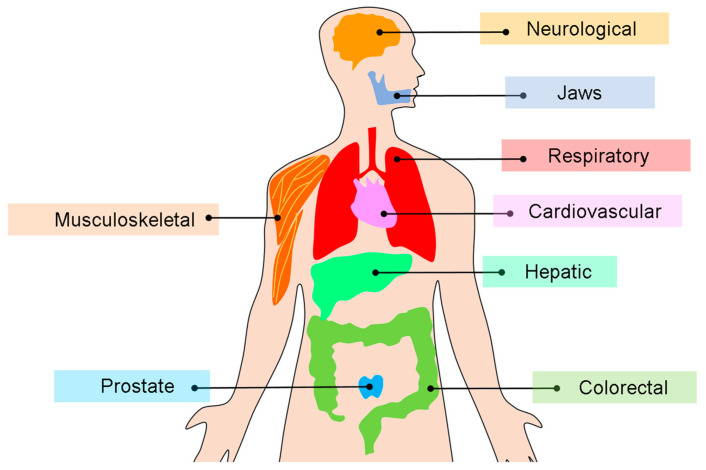
Clinical applications of radiomics in cross-sectional imaging modalities. The diagram illustrates the principal organ systems and disease domains to which radiomics has been applied. Oncologic applications of radiomics include lung, head and neck, colorectal, and prostate cancers. Beyond oncology, radiomics has been increasingly explored in cardiology (e.g., coronary plaque and myocardial characterization), neurology (e.g., stroke and neurodegenerative disorders), musculoskeletal imaging (e.g., bone and joint assessment), and dentomaxillofacial radiology (e.g., jaw lesions and temporomandibular joint evaluation). Together, these examples highlight the expanding role of radiomics in both cancer and non-cancer settings, thus leveraging CT, MRI, PET/CT, and CBCT. Adapted from McCague et al. [27].

Figure 5 shows a visual overview of organ systems in which radiomics has been applied to cross-sectional imaging, illustrating both oncologic and non-oncologic domains.

## 10. Validation of Radiomics Across Imaging Modalities: CT, CBCT PET/CT, and MRI

Radiomics validation has been extensively investigated across multiple imaging modalities, each offering unique advantages for quantitative feature extraction [1,5]. As for MRI, radiomics benefits from superior soft-tissue contrast, which enables sensitive detection of tumor heterogeneity and microenvironmental changes [13,39]. However, reproducibility is influenced by variations in acquisition parameters, magnetic field strength, and pulse sequences, requiring harmonization strategies and standardized preprocessing to ensure reliable feature extraction [39,41,54].

As for CT, radiomics has been validated more consistently due to the high spatial resolution and standardized voxel intensity scale (Hounsfield units) of this modality. CT radiomics has demonstrated prognostic value in lung, head and neck, and abdominal cancers, with features such as texture and shape descriptors proving to be robust in multicenter datasets. Still, variability in reconstruction kernels and acquisition protocols can impact reproducibility, which highlights the importance of protocol harmonization and phantom studies for validation [35,36,37,48].

PET/CT contributes with complementary metabolic information to anatomical imaging, in which radiomic features are derived from standardized uptake values (SUV) and texture matrices have strong potential for outcome prediction and treatment response monitoring. Validation studies have emphasized the importance of harmonized acquisition protocols, reconstruction parameters, and intensity discretization methods, as these strongly affect the reproducibility of PET-based features. Multicenter initiatives such as the IBSI have played a critical role in enhancing consistency in the PET/CT radiomics research [39,41,54].

Lately, CBCT has emerged as a valuable platform for radiomics, particularly in adaptive radiotherapy and dentomaxillofacial imaging. Although CBCT images typically exhibit more scatter and motion artifacts than diagnostic CT, studies have demonstrated that reproducible radiomic features can indeed be extracted under controlled conditions [11,33,34,50,51,53]. For example, Fave et al. [49] reported that a substantial subset of texture features reached high reproducibility (concordance correlation coefficient > 0.9) when acquisition protocols were consistent and patient motion was limited.

A key point is that CBCT’s routine use in image-guided radiotherapy and dental practice provides unique opportunities for longitudinal and low-dose radiomic applications, where relative changes in features across time points may serve as biomarkers of treatment response or disease progression [11,33,34,50,51,53].

Taken together, validation across MRI, CT, PET/CT, and CBCT underscores the need for a modality-specific optimization while pursuing cross-platform reproducibility. Although MRI and CT remain being the most standardized in the radiomics research, PET/CT introduces valuable functional dimensions and CBCT establishes itself as a promising tool with growing evidence of reproducible feature extraction. Taken together, robust validation across all modalities (through test–retest analyses, phantom experiments, and multicenter clinical trials) is essential to translate radiomics into clinically reliable and generalizable imaging biomarkers [29,30,37].

In parallel, AI has become a key enabler of this process. Machine learning (ML) techniques refine feature selection and build predictive models, whereas deep learning allows automatic extraction of complex image representations. Together, these approaches enhance performance and accelerate the transition of radiomics from research to routine clinical practice [70].

ML plays a central role in radiomics by selecting the most informative features from high-dimensional datasets and by using them to train predictive or prognostic models [62,70]. Common algorithms include support vector machines, random forests, gradient boosting, and regularized logistic regression, all of which can classify lesions, predict treatment response, or stratify patient risk [63,64,65,66,67]. By reducing redundancy and focusing on relevant features, ML improves model stability and interpretability, making it a key enabler in the transition of radiomics from exploratory research to clinically applicable decision-support tools [64,67].

Recent systematic reviews and meta-analyses have further emphasized the need for multicenter validation to ensure the generalizability of radiomics models. Initiatives such as the IBSI and multicenter studies from the Radiomics Quality Score (RQS) framework have provided important methodological benchmarks for reproducibility and transparency. In parallel, imaging challenges organized by international consortia, including MICCAI (Medical Image Computing and Computer-Assisted Intervention) and the RSNA (Radiological Society of North America) AI Challenges, have enabled independent evaluation of algorithms on shared, annotated datasets. These competitions have revealed both the potential and the limitations of current radiomic approaches—particularly issues related to data harmonization, feature robustness, and model overfitting—offering valuable insights into the translation of AI-based radiomics into clinical practice [29,30,37,83].

Despite the growing number of radiomics publications, the rate of independent external validation remains limited, often restricting model generalizability across institutions and imaging protocols. Differences in scanners, reconstruction algorithms, and acquisition settings can introduce batch effects and domain shifts, leading to substantial variations in feature distributions. To address these challenges, several best practices have been proposed, including ComBat harmonization, phantom calibration, and cross-center data normalization, all of which aim to preserve biological signal while minimizing technical bias [29,30,36,37]. Additionally, public benchmarking platforms and open challenges—such as those promoted by IBSI and MICCAI (Medical Image Computing and Computer-Assisted Intervention)—are essential for independent performance evaluation. Future studies should incorporate systematic model auditing, transparent reporting (e.g., Radiomics Quality Score, RQS), and publicly available code and datasets to strengthen the robustness and reproducibility of radiomics-based models [37,40,41,83].

## 11. Integrating Radiomics into the Radiologist’s Report

The clinical translation of radiomics depends not only on robust methodological development, but also on its integration into a radiologist’s daily workflow. For radiomics to become a practical tool rather than a purely research-oriented concept, its features and predictive models must be incorporated into the reporting process [84].

### 11.1. From Descriptive to Quantitative Reporting

Traditional radiology reports rely primarily on qualitative descriptions of lesion size, morphology, enhancement, and location [85]. Radiomics enables a shift toward quantitative radiology, in which imaging biomarkers extracted from CT, MRI, CBCT, or PET scans are incorporated into the report along with conventional findings [1,3]. This integration transforms the radiologist’s role from a purely descriptive observer into a provider of quantitative and reproducible biomarkers. For example, instead of reporting only “a heterogeneous mass with irregular margins,” a radiologist could add radiomic-derived measures of texture heterogeneity or shape descriptors, linking them to prognosis or likelihood of treatment response [4,8].

### 11.2. Decision Support and Predictive Models

Radiomics can be applied in real time to assist radiologists in refining differential diagnoses, staging criteria, and risk stratification. Predictive and prognostic models derived from radiomic signatures may be integrated into PACS/RIS systems or reporting software. This offers automated outputs, such as the probability of malignancy for a pulmonary nodule, a radiomic risk score for treatment response in head and neck cancer, or a quantitative heterogeneity index in musculoskeletal and cardiovascular diseases [1,4,5,8].

### 11.3. Structured Reporting and Standardization

To be clinically valuable, radiomic information should be communicated by means of structured reporting templates. These may include specific fields for quantitative features, prognostic models, and AI-assisted outputs. Adoption of international guidelines, such as the IBSI, is critical to ensure that radiomic biomarkers reported by different centers are comparable and reproducible [1,9,86,87].

### 11.4. Practical Challenges

Several barriers remain before radiomics can be fully integrated into radiology reporting. First, workflow integration is essential as radiomic extraction and analysis must be automated and embedded into existing PACS systems to avoid additional workload [1,88]. Second, interpretability represents a challenge as radiologists must be trained to understand which radiomic features are clinically meaningful and how they relate to the pathology and patient outcomes [41,71]. Third, only radiomic biomarkers validated across multicenter datasets and regulatory frameworks should be incorporated into the report, thus ensuring reliability and generalizability [9,83]. Finally, effective communication with clinicians is decisive, as radiomic results must be presented in a way that is both understandable and actionable for referring physicians, without overwhelming them with unnecessary detail [5,72,89,90].

### 11.5. Regulatory Perspectives

In recent years, regulatory agencies such as the FDA (U.S. Food and Drug Administration) and CE (Conformité Européenne) have begun establishing dedicated pathways for the approval of AI- and radiomics-based medical software, classifying them as Software as a Medical Device (SaMD). These frameworks require demonstration of analytical validity, clinical performance, and continuous post-market monitoring. In parallel, standardization initiatives such as the IBSI and quality assessment tools like the Radiomics Quality Score (RQS) provide essential methodological guidance to align research outputs with regulatory expectations. Such harmonization efforts are critical to ensure that radiomics models can transition from research prototypes to clinically certified tools that meet safety, reproducibility, and interoperability standards [9,40,41,71].

## 12. Current Landscape: Research or Routine?

Despite growing enthusiasm, the incorporation of radiomics into the everyday practice of radiology is still limited [5]. At present, radiomics is predominantly used in research settings, particularly in oncology, where it has been studied for screening, disease detection, staging, prognosis, and treatment response assessment [6]. In specialized research centers and clinical trials, radiomics-based biomarkers are being piloted to stratify patients and complement conventional staging [91].

However, several factors delay its widespread adoption: lack of standardized imaging protocols and feature extraction pipelines [9,87], variability across scanners and centers [36,37], limited reproducibility in external cohorts [29,30], and absence of seamless integration into PACS/RIS environments [71]. Moreover, most radiomic models remain insufficiently interpretable for routine use, which hinders trust among radiologists and clinicians [63].

Professional initiatives, such as IBSI [9] and practice recommendations from professional societies (e.g., European Society of Medical Imaging Informatics) [89], are helping address these challenges to promote reproducibility and standardization. As machine learning and AI mature, radiomics is gradually moving toward translation into routine workflows [70,84].

## 13. Future Perspectives

The ultimate vision is a radiomics-augmented radiology report, in which conventional findings are enriched by validated imaging biomarkers and predictive signatures [1,5,84,91]. Such reports would not replace the expertise of radiologists, but rather enhance their interpretive power by enabling objective, reproducible, and personalized insights. Although radiomics today remains largely confined to the research domain, its trajectory suggests that its integration into daily practice is imminent, provided that reproducibility, workflow integration, and interpretability challenges are adequately resolved.

Looking ahead, AI-driven self-updating models may enable radiomic signatures to continuously adapt as new data become available, enhancing their robustness and clinical utility. In parallel, federated learning approaches, where models are trained across institutions without direct data sharing, represent a promising strategy to overcome privacy and regulatory barriers to multicenter collaboration [62,71,72]. These innovations could accelerate the translation of radiomics into routine workflows while maintaining compliance with data protection standards.

## Figures and Tables

**Table 1:** Comparison of Selected Radiomics Tools.

	License		Primary Modalities					Segmentation Tool		Segmentation Type		
	Open	Commercial	CBCT	CT	MRI	PET	Radiotherapy	ROI	VOI	Manual	Semi-Automatic	Automatic
MaZda												
PyRadiomics												
LIFEx												
MATLAB												
IBEX												
RaCaT												
CERR												
RadiomiX												

Cells highlighted in gray indicate that the feature is available in the corresponding radiomics tool, while blank cells indicate that the feature is not supported or not primarily implemented.

**Table 2:** Comparison of Selected Radiomics features, advantages and limitations.

	Key Features	Advantages	Limitations
MaZda	GUI-based texture analysis (GLCM, GLRLM, histogram)	Easy to use; ideal for education and historical comparisons	No built-in preprocessing; no DICOM integration; dated interface
PyRadiomics	IBSI-compliant features; preprocessing (resampling, normalization); 3D Slicer integration	Highly flexible; supports batch processing; large community	Requires coding for advanced customization
LIFEx	Built-in segmentation; phantom calibration; IBSI-compliant	User-friendly; minimal coding; DICOM import	Limited custom feature development; PET/CT focus
MATLAB	Image Processing Toolbox; ML toolbox; custom feature design	Highly flexible; supports pipelines; AI integration	Requires license; programming skills needed
IBEX	GUI ROI/VOI segmentation; multimodal analysis; feature extraction	Versatile; MATLAB extensions supported	Dependent on MATLAB; development limited
RaCaT	Batch processing; multicenter harmonization	Good for large-scale multicenter studies	Narrower feature set compared to PyRadiomics
CERR	Dose-volume histogram; radiomics from planning images	Ideal for radiotherapy datasets; RT integration	MATLAB dependency; focused on RT workflow
RadiomiX	Regulatory-grade; QA/QC modules; DICOM integration	CE-marked; robust clinical validation	Cost; closed-source

## Data Availability

No new data were created or analyzed in this study.
